# Aryl derivatives of 3H-1,2-benzoxathiepine 2,2-dioxide as carbonic anhydrase inhibitors

**DOI:** 10.1080/14756366.2019.1695795

**Published:** 2019-12-02

**Authors:** Aleksandrs Pustenko, Alessio Nocentini, Anastasija Balašova, Ahmed Alafeefy, Mikhail Krasavin, Raivis Žalubovskis, Claudiu T. Supuran

**Affiliations:** a Latvian Institute of Organic Synthesis, Riga, Latvia; b Institute of Technology of Organic Chemistry, Faculty of Materials Science and Applied Chemistry, Riga Technical University, Riga, Latvia; c Dipartimento Neurofarba, Sezione di Scienze Farmaceutiche e Nutraceutiche, Universita degli Studi di Firenze, Florence, Italy; d Faculty of Pharmacy, University Technology MARA, UiTM, Bandar, Malaysia; e Chemistry Department, Saint Petersburg State University, Saint Petersburg, Russian Federation

**Keywords:** Carbonic anhydrase, transmembrane isoforms, sulfocoumarin, homosulfocoumarin, isoform-selective inhibitor

## Abstract

A new series of homosulfocoumarins (3H-1,2-benzoxathiepine 2,2-dioxides) possessing various substitution patterns and moieties in the 7, 8 or 9 position of the heterocylic ring were prepared by original procedures and investigated for the inhibition of four physiologically relevant carbonic anhydrase (CA, EC 4.2.1.1) isoforms, the human (h) hCA I, II, IX and XII. The 8-substituted homosulfocoumarins were the most effective hCA IX/XII inhibitors followed by the 7-substituted derivatives, whereas the substitution pattern in position 9 led to less effective binders for the transmembrane, tumour-associated isoforms IX/XII. The cytosolic isoforms hCA I and II were not inhibited by these compounds, similar to the sulfocoumarins/coumarins investigated earlier. As hCA IX and XII are validated anti-tumour targets, with one sulphonamide (SLC-0111) in Phase Ib/II clinical trials, finding derivatives with better selectivity for inhibiting the tumour-associated isoforms over the cytosolic ones, as the homosulfocoumarins reported here, is of crucial importance.

## 1. Introduction

Carbonic anhydrases (CAs, EC 4.2.1.1) are metalloenzymes widespread in nature, being encoded by at least eight different genetic families, which have been identified in organisms all over the phylogenetic tree^1–3^. By catalysing a crucial physiologic reaction, by which CO_2_ is hydrated with the formation of a weak base (bicarbonate) and a strong acid (hydronium ions), these enzymes are involved in a multitude of physiologic processes, starting with pH regulation and ending with metabolism^1^^,^^3–6^. As thus, CAs are drug targets for decades, with their inhibitors having pharmacological applications in a multitude of fields^1^^,^^3–5^. The primary sulphonamides were discovered as CA inhibitors (CAIs) in the 40 s, and most of the drugs that were launched in the next decades as diuretics, antiepileptics, or antiglaucoma agents targeting CAs belonged to this class of compounds^1^^,^^3–5^. Although highly effective as CAIs^1^, the sulphonamides generally indiscriminately inhibit most α-CA isoforms present in mammals (at least 15 in humans, and 16 in other vertebrates^1^) as well as CAs belonging to the other genetic families (β-, γ-, δ-, ζ-, η-, θ- and ι-CAs)^2–5^ and for this reason alternative CAI classes were searched for. In fact, in the last 10 years, a multitude of new chemotypes as well as novel CA inhibition mechanisms were reported^1^^,^^4^^,^^7–9^, which highly enriched our understanding of these enzymes and also allowed for obtaining isoform-selective CAIs targeting all the mammalian isoforms^4^^,^^7–9^. Among the new such chemotypes, which also showed the highest levels of isoform selectivity, were the coumarins^9^, the sulfocoumarins^7^^,^^8^ and their congeners, homosulfocoumarins (3H-1,2-benzoxathiepine 2,2-dioxides)^10^. Considering the fact that this last chemotype was only recently reported and rather poorly investigated^10^, we report here a series of new aryl-3H-1,2-benzoxathiepine 2,2-dioxides substituted in various positions of the heterocyclic ring, which have been designed in order to explore the chemical space around this new CA inhibitory chemotype and to see whether the presence of various moieties in position 7, 8 or 9 of the heterocyclic system maintains the desired enzyme inhibitory activity and selectivity for the target isoforms.

## 2. Materials and methods

### 2.1. Chemistry

Reagents, starting materials and solvents were obtained from commercial sources and used as received. Thin-layer chromatography was performed on silica gel, spots were visualised with UV light (254 and 365 nm). Melting points were determined on an OptiMelt automated melting point system. IR spectra were recorded on Shimadzu FTIR IR Prestige-21 spectrometer. NMR spectra were recorded on Bruker Advance Neo (400 MHz) spectrometer with chemical shifts values (δ) in ppm relative to TMS using the residual DMSO-d_6_ signal (^1^H 2.50; ^13 ^C 39.52) or CDCl_3_ signal (^1^H 7.26; ^13 ^C 77.16) as an internal standard. High-resolution mass spectra (HRMS) were recorded on a mass spectrometer with a Q-TOF micro mass analyser using the ESI technique. Elemental analyses were measured using Carlo Erba (EA1108) apparatus (Milan, Italy).

#### 2-Hydroxy-5-iodobenzaldehyde (2)


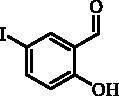
To a solution of salicylaldehyde (**1**) (8.73 mL, 81.9 mmol) in AcOH (40 mL) iodine monochloride (4.92 mL, 98.3 mmol) was added^11^. Reaction mixture was stirred 24 h at 40 °C, then cooled to r.t. EtOH (60 mL) was added and all volatiles were removed in vacuum. CH_2_Cl_2_ (60 mL) and water (100 mL) were added, the phases were separated and the aqueous phase was extracted with CH_2_Cl_2_ (3 × 50 mL). The combined organic phases were washed with 10% Na_2_S_2_O_3_ (1 × 60 mL), brine (1 × 60 mL), dried over Na_2_SO_4_, filtered and concentrated. The residue was purified by column chromatography on silica gel (PE/EtOAc 3:1), the crude product was re-crystallised from EtOH to afford product **2** (17.1 g, 84%) as yellowish solid. ^1^H NMR (400 MHz, DMSO-d_6_) δ = 6.85 (d, 1H, *J* = 8.6 Hz), 7.77 (dd, 1H, *J* = 8.6, 2.4 Hz), 7.87 (d, 1H, *J* = 2.4 Hz), 10.16 (s, 1H), 10.92 (s, 1H) ppm. ^13 ^C NMR (100 MHz, DMSO-d_6_) δ = 81.4, 120.1, 124.6, 136.7, 144.1, 160.3, 189.8 ppm.

#### Prop-2-ene-1-sulphonyl chloride (4)


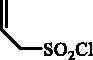
Compound was synthesised using previously described procedure by our group^10^. To a solution of Na_2_SO_3_ (30.2 g; 0.24 mol) in water (140 mL) ally bromide (17.4 mL; 0.20 mol) was added and the reaction mixture was refluxed overnight. After cooling to room temperature, reaction mixture was washed with Et_2_O (3 × 50 mL). Aqueous phase was concentrated. Crude white solid was dried under high vacuum at 100 °C for 6 h. To the white solid at 0 °C POCl_3_ (120 mL) was added, and mixture was refluxed for 4 h. After cooling to room temperature dry THF (60 mL) was added and reaction mixture was vigorously stirred for 10 min and filtered. Filter cake was suspended in dry THF (60 mL), suspension was vigorously stirred for 10 min and filtered. Filtrates were combined and solvent was carefully driven off on rotary evaporator. Residue was distilled in vacuum (10 mbar) and fraction with boiling point 38–42 °C was collected, to give prop-2-ene-1-sulfonil chloride (**4**) as colourless oil (18.6 g, 66%), which was used in further reactions without additional purification.

#### General procedure for the synthesis of ethenylphenoles (3, 14, 18, 27)

To a stirred solution of methyltriphenylphosphonium bromide (2.60 eq) in dry THF (5 mL/1 mmol of methyltriphenylphosphonium bromide), was added tBuOK (3.2 eq) in several portions over 20 min. Reaction mixture was stirred for 1 h at r.t. Corresponding benzaldehyde (1 eq) was added and stirring continued at room temperature for 24 h. Reaction mixture was diluted with CH_2_Cl_2_ (4 mL/1 mmol of methyltriphenylphosphonium bromide). Organic layer was washed with water (2 × 20 mL) and brine (2 × 20 mL), and dried over Na_2_SO_4_, filtered and concentrated. The crude product was purified by column chromatography on silica gel (PE/EtOAc 4:1).

#### 4-Iodo-2-ethenylphenol (3)


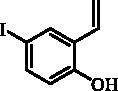
Compound **3** was prepared according to the general procedure from methyltriphenylphosphonium bromide (14.98 g, 37.0 mmol), *t*-BuOK (5.79 g, 51.6 mmol) and 2-hydroxy-5-iodobenzaldehyde (**2**) (4.00 g, 16.1 mmol) as yellowish solid (3.29 g, 83%)^12^. ^1^H NMR (400 MHz, DMSO-d_6_) δ = 5.23 (dd, 1H, *J* = 11.3, 1.4 Hz), 5.80 (dd, 1H, *J* = 17.8, 1.4 Hz), 6.67 (d, 1H, *J* = 8.6 Hz), 6.77–6.87 (m, 1H), 7.38 (dd, 1H, *J* = 8.5, 2.3 Hz), 7.70 (d, 1H, *J* = 2.3 Hz), 9.94 (s, 1H) ppm ^13 ^C NMR (100 MHz, DMSO-d_6_) δ = 81.4, 115.1, 118.4, 126.9, 130.4, 134.4, 137.0, 154.6 ppm.

#### 3-Bromo-2-ethenylphenol (14)


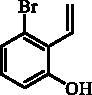
Compound **14** was prepared according to the general procedure from methyltriphenylphosphonium bromide (18.48 g; 51.7 mmol), *t*-BuOK (7.15 g; 63.7 mmol) and 2-bromo-5-hydroxybenzaldehyde (**13**) (4.00 g, 19.9 mmol) as yellowish solid (3.25 g; 82%). ^1^H NMR (400 MHz, DMSO-d_6_) δ = 5.51 (dd, 1H, *J* = 12.0, 2.4 Hz), 6.06 (dd, 1H, *J* = 17.7, 2.4 Hz), 6.76 (dd, 1H, *J* = 17.7, 11.9 Hz), 6.86–6.91 (m, 1H), 6.98 (t, 1H, *J* = 8.0 Hz), 7.07 (dd, 1H, *J* = 8.0, 1.2 Hz), 10.18 (s, 1H) ppm ^13 ^C NMR (100 MHz, DMSO-d_6_) δ = 115.4, 120.9, 123.2, 123.4, 124.2, 129.1, 132.2, 157.1 ppm.

#### 5-Bromo-2-ethenylphenol (18)


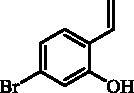
Compound **18** was prepared according to the general procedure from methyltriphenylphosphonium bromide (18.48 g; 51.7 mmol), *t*-BuOK (7.15 g; 63.7 mmol) and 4-bromo-2-hydroxybenzaldehyde (**17**) (4.00 g, 19.9 mmol) as yellowish solid (3.01 g; 76%)^13^. ^1^H NMR (400 MHz, DMSO-d_6_) δ = 5.24 (dd, 1H, *J* = 11.3, 1.6 Hz), 5.79 (dd, 1H, *J* = 17.8, 1.6 Hz), 6.86 (dd, 1H, *J* = 17.8, 11.3 Hz), 6.93–6.97 (m, 1H), 7.00 (d, 1H, *J* = 2.0 Hz), 7.37 (d, 1H, *J* = 8.3 Hz), 10.13 (s, 1H) ppm ^13 ^C NMR (100 MHz, DMSO-d_6_) δ = 114.6, 118.3, 120.8, 122.0, 123.5, 128.0, 130.8, 155.7 ppm.

#### 2-Bromo-6-ethenylphenol (27)


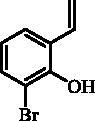
Compound **27** was prepared according to the general procedure from methyltriphenylphosphonium bromide (18.48 g; 51.7 mmol), *t*-BuOK (7.15 g; 63.7 mmol) and 3-bromo-2-hydroxybenzaldehyde (**26**) (4.00 g, 19.9 mmol) as yellowish solid (3.17 g; 80%)^14^.

^1^H NMR (400 MHz, DMSO-d_6_) δ = 5.29 (dd, 1H, *J* = 11.2, 1.3 Hz), 5.78 (dd, 1H, *J* = 17.6, 1.4 Hz), 6.80 (t, 1H, *J* = 7.8 Hz), 7.02 (dd, 1H, *J* = 17.6, 11.2 Hz), 7.41–7.49 (m, 2H), 9.32 (s, 1H) ppm ^13 ^C NMR (100 MHz, DMSO-d_6_) δ = 112.2, 115.5, 121.3, 125.4, 127.5, 131.4, 132.1, 150.7 ppm.

#### General procedure for diolefine (5, 15, 19, 28) synthesis

To a stirred solution of corresponding ethenylphenol (**3, 14, 18, 27**) (1 eq) in CH_2_Cl_2_ (10 mL/1 mmol corresponding ethenylphenol) at 0 °C was added prop-2-ene-1-sulphonyl chloride (**4**) (1.39 eq) and Et_3_N (1.4 eq). Reaction mixture was stirred overnight (20 h) at room temperature. Water (30 mL) was added, reaction mixture was extracted with EtOAc (3 × 40 mL), combined organic extracts were washed with brine (2 × 40 mL), and dried over dried over Na_2_SO_4_, filtered and concentrated. The crude product was purified by column chromatography on silica gel (EtOAc/PE 1:4).

#### 4-Iodo-2-ethenylphenyl prop-2-ene-1-sulfonate (5)


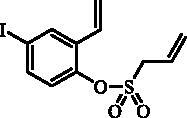
Compound **5** was prepared according to the general procedure from 4-iodo-2-ethenylphenol (**3**) (2.00 g; 8.13 mmol), prop-2-ene-1-sulphonyl chloride (**4**) (1.11 mL; 10.57 mmol) and NEt_3_ (1.58 mL; 11.38 mmol) as yellowish oil (2.36 g; 83%). IR (film, cm^−1^) *ν*_max_= 1373 (S = O), 1160 (S = O). ^1^H NMR (400 MHz, DMSO-d_6_) δ = 4.46–4.50 (m, 2H), 5.44–5.55 (m, 2H), 5.56–5.63 (m, 1H), 5.85–5.97 (m, 1H), 6.02 (d, 1H, *J* = 17.6 Hz), 6.84 (dd, 1H, *J* = 17.8, 11.2 Hz), 7.15 (d, 1H, *J* = 8.6 Hz), 7.72 (dd, 1H, *J* = 8.6, 2.2 Hz), 8.10 (d, 1H, *J* = 2.2 Hz) ppm. ^13 ^C NMR (100 MHz, DMSO-d_6_) δ = 54.9, 93.0, 119.0, 124.6, 125.0, 125.3, 128.4, 133.1, 134.9, 137.9, 145.7 ppm. HRMS (ESI) [M + H]^+^: *m*/*z* calcd for C_11_H_12_O_3_SI: 350.9552. Found 350.9542.

#### 3-Bromo-2-vinylphenyl prop-2-ene-1-sulfonate (15)


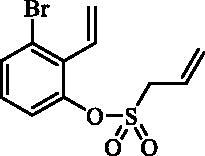
Compound **15** was prepared according to the general procedure from 3-bromo-2- ethenylphenol (**14**) (2.00 g; 10.05 mmol), prop-2-ene-1-sulphonyl chloride (**4**) (1.37 mL; 13.06 mmol) and NEt_3_ (1.96 mL; 14.07 mmol) as yellowish oil (2.01 g; 66%). IR (film, cm^−1^) *ν*_max_= 1368 (S = O), 1174 (S = O), 1160 (S = O).

^1^H NMR (400 MHz, DMSO-d_6_) δ = 4.41 (dt, 2H, *J* = 7.2, 1.0 Hz), 5.49–5.53 (m, 1H), 5.55–5.61 (m, 1H), 5.69–5.76 (m, 2H), 5.83–5.94 (m, 1H), 6.63 (dd, 1H, *J* = 17.9, 11.7 Hz), 7.30–7.35 (m, 1H), 7.43–7.46 (m, 1H), 7.67 (dd, 1H, *J* = 8.0, 1.1 Hz) ppm. ^13 ^C NMR (100 MHz, DMSO-d_6_) δ = 55.4, 122.4, 123.5, 123.7, 124.5, 125.2, 129.8, 130.6, 131.6, 131.9, 146.9 ppm. HRMS (ESI) [M + H]^+^: *m*/*z* calcd for C_11_H_12_O_3_SBr: 302.9691. Found 302.9681.

#### 5-Bromo-2-vinylphenyl prop-2-ene-1-sulfonate (19)


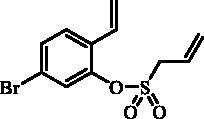
Compound **19** was prepared according to the general procedure from 5-bromo-2- ethenylphenol (**18**) (2.00 g; 10.05 mmol), prop-2-ene-1-sulphonyl chloride (**4**) (1.37 mL; 13.06 mmol) and NEt_3_ (1.96 mL; 14.07 mmol) as yellowish oil (1.65 g; 54%). IR (film, cm^−1^) *ν*_max_= 1377 (S = O), 1161 (S = O). ^1^H NMR (400 MHz, DMSO-d_6_) δ = 4.54 (dt, 2H, *J* = 7.2, 1.0 Hz), 5.48 (dd, 1H, *J* = 11.2, 0.8 Hz), 5.52–5.56 (m, 1H), 5.58–5.64 (m, 1H), 5.86–5.98 (m, 1H), 5.99 (dd, 1H, *J* = 17.6, 0.9 Hz), 6.89 (dd, 1H, *J* = 17.8, 11.2 Hz), 7.55–7.59 (m, 2H), 7.73–7.77 (m, 1H) ppm. ^13 ^C NMR (100 MHz, DMSO-d_6_) δ = 55.1, 118.4, 120.8, 124.5, 125.4, 125.6, 128.1, 128.7, 130.3, 130.5, 146.1 ppm. HRMS (ESI) [M + H]^+^: *m*/*z* calcd for C_11_H_12_O_3_SBr: 302.9691. Found 302.9684.

#### 2-Bromo-6-vinylphenyl prop-2-ene-1-sulfonate (28)


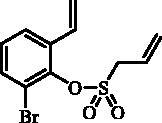
Compound **28** was prepared according to the general procedure from 2-bromo-6-ethenylphenol (**27**) (2.00 g; 10.05 mmol), prop-2-ene-1-sulphonyl chloride (**4**) (1.37 mL; 13.06 mmol) and NEt_3_ (1.96 mL; 14.07 mmol) as yellowish oil (2.62 g; 86%). IR (film, cm^−1^) *ν*_max_= 1367 (S = O), 1179 (S = O), 1165 (S = O). ^1^H NMR (400 MHz, CDCl_3_) δ = 4.31 (dt, 2H, *J* = 7.2, 1.0 Hz), 5.45 (dd, 1H, *J* = 11.0, 0.80 Hz), 5.57–5.65 (m, 2H), 5.81 (dd, 1H, *J* = 17.5, 0.8 Hz), 6.03–6.15 (m, 1H), 7.07–7.17 (m, 2H), 7.52–7.59 (m, 2H) ppm. ^13 ^C NMR (100 MHz, CDCl_3_) δ = 57.9, 117.7, 118.4, 123.9, 125.7, 126.0, 128.3, 130.9, 133.1, 135.1, 144.4 ppm. HRMS (ESI) [M + H]^+^: *m*/*z* calcd for C_11_H_12_O_3_SBr: 302.9691. Found 302.9681.

#### General method for 3*H*-1,2-benzoxathiepine 2,2-dioxide halogen derivative (7, 20, 29) synthesis

To a solution of corresponding diolefine (**5, 15, 19, 28**) (1.0 eq) in dry, degassed toluene (15 mL/1 mmol corresponding diolefine) ruthenium catalyst **6** (5 mol %) was added. Reaction mixture was bubbled with argon for 5 min and sealed, stirred at 70 °C for 4 h. After cooling to r.t. it was concentrated, and the crude product was purified by column chromatography on silica gel (EtOAc/PE 1:4). Products were re-crystallised from EtOH.

#### 7-Iodo-3*H*-1,2-benzoxathiepine 2,2-dioxide (7)


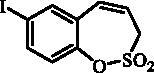
Compound **7** was prepared according to the general procedure from diolefine (**5**) (1.00 g; 2.86 mmol) and ruthenium catalyst **6** (0.14 g; 0.14 mmol) as yellowish solid (0.82 g; 89%). Mp 127–128 °C. IR (film, cm^−1^) *ν*_max_= 1370 (S = O), 1164 (S = O), 1155 (S = O). ^1^H NMR (400 MHz, DMSO-d_6_) δ = 4.52 (dd, 2H, *J* = 5.8, 1.3 Hz), 5.97–6.04 (m, 1H), 6.82–6.87 (m, 1H), 7.14 (d, 1H, *J* = 8.5 Hz), 7.79 (dd, 1H, *J* = 8.5, 2.2 Hz), 7.88 (d, 1H, *J* = 2.2 Hz) ppm. ^13 ^C NMR (100 MHz, DMSO-d_6_) δ = 51.6, 92.3, 121.5, 124.5, 129.8, 130.4, 138.7, 139.6, 146.7 ppm. Anal. Calcd for C_9_H_7_IO_3_S: C, 33.56; H, 2.19. Found: C, 33.55; H, 2.21.

#### 8-Bromo-3*H*-1,2-benzoxathiepine 2,2-dioxide (20)


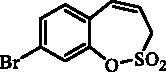
Compound **20** was prepared according to the general procedure from diolefine (**19**) (1.23 g; 4.06 mmol) and ruthenium catalyst **6** (0.19 g; 0.20 mmol) as white solid (1.0 g; 90%). Mp 144–145 °C. IR (film, cm^−1^) *ν*_max_= 1359 (S = O), 1182 (S = O), 1165 (S = O). ^1^H NMR (400 MHz, DMSO-d_6_) δ = 4.54 (dd, 2H, *J* = 5.8, 1.0 Hz), 5.95–6.05 (m, 1H), 6.87 (d, 1H, *J* = 11.4 Hz), 7.42–7.47 (m, 1H), 7.58–7.66 (m, 2H) ppm. ^13 ^C NMR (100 MHz, DMSO-d_6_) δ = 51.9, 120.9, 122.0, 125.2, 127.5, 130.1, 130.3, 133.0, 147.1 ppm. Anal. Calcd for C_9_H_7_BrO_3_S: C, 39.29; H, 2.56. Found: C, 39.28; H, 2.59.

#### 9-Bromo-3*H*-1,2-benzoxathiepine 2,2-dioxide (29)


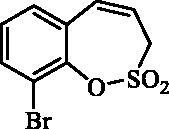
Compound **29** was prepared according to the general procedure from diolefine (**28**) (2.20 g; 7.26 mmol) and ruthenium catalyst **6** (0.34 g; 0.36 mmol) as yellowish solid (1.55 g; 78%). Mp 113–114 °C. IR (film, cm^−1^) *ν*_max_= 1364 (S = O), 1177 (S = O). ^1^H NMR (400 MHz, CDCl_3_) δ = 4.10 (dd, 2H, *J* = 6.0, 1.2 Hz), 5.95–6.03 (m, 1H), 6.82–6.87 (m, 1H), 7.18 (t, 1H, *J* = 7.8 Hz), 7.24–7.28 (m, 1H), 7.66 (dd, 1H, *J* = 7.9, 1.6 Hz) ppm. ^13 ^C NMR (100 MHz, CDCl_3_) δ = 51.8, 117.7, 120.1, 128.0, 130.0, 130.1, 132.2, 134.2, 144.9 ppm. Anal. Calcd for C_9_H_7_BrO_3_S: C, 39.29; H, 2.56. Found: C, 39.28; H, 2.58.

#### General method for 3*H*-1,2-benzoxathiepine 2,2-dioxide aril derivative (8–12, 21–25 and 30–34) synthesis

In a pressure tube corresponding 3*H*-1,2-benzoxathiepine 2,2-dioxide halogen derivative (**7, 20, 29**) (1.0 eq) was dissolved in dry toluene (6 mL/1 mmol corresponding 3*H*-1,2-benzoxathiepine 2,2-dioxide halogen derivative), degassed water was added (5% from toluene volume), corresponding boronic acid (1.5 eq), K_3_PO_4_ (2.0 eq) and Pd(PPh_3_)_4_ (0.1 eq). Reaction mixture was bubbled with argon 5 min, tube was sealed and heated for 16 h at 100 °C temperature. Reaction mixture was cooled to r.t., filtered through cellite was washed with EtOAc (40 mL). Mixture was evaporated and crude product was purified by column chromatography on silica gel (EtOAc/PE 1:3). Products were re-crystallised from EtOH.

#### 7- Phenyl-3*H*-1,2-benzoxathiepine 2,2-dioxide (8)


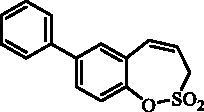
Compound **8** was prepared according to the general procedure from 7-iodo-3*H*-1,2-benzoxathiepine 2,2-dioxide (**7**) (0.20 g; 0.62 mmol) phenylboronic acid (0.11 g; 0.93 mmol), K_3_PO_4_ (0.26 g; 1.24 mmol) and Pd(PPh_3_)_4_ (72 mg; 0.062 mmol) as white solid (95 mg; 56%). Mp 144–145 C. IR (film, cm^−1^) *ν*_max_=1366 (S = O), 1363 (S = O), 1172 (S = O), 1164 (S = O). ^1^H NMR (400 MHz, CDCl_3_) δ = 4.06 (dd, 2H, *J* = 6.2, 0.8 Hz), 5.99–6.07 (m, 1H), 6.95 (d, 1H, *J* = 11.0 Hz), 7.38–7.43 (m, 2H), 7.44–7.52 (m, 3H), 7.54–7.58 (m, 2H), 7.62 (dd, 1H, *J* = 8.4, 2.2 Hz) ppm. ^13 ^C NMR (100 MHz, CDCl_3_) δ = 51.4, 119.8, 123.3, 127.3, 128.1, 128.5, 129.1, 129.3, 129.4, 132.9, 139.4, 140.6, 147.1 ppm. Anal. Calcd for C_15_H_12_O_3_S: C, 66.16; H, 4.44.Found: C, 66.06; H, 4.45.

#### 7–(4-Methoxyphenyl)-3*H*-1,2-benzoxathiepine 2,2-dioxide (9)


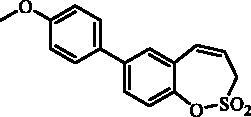
Compound **9** was prepared according to the general procedure from 7-iodo-3*H*-1,2-benzoxathiepine 2,2-dioxide (**7**) (0.20 g; 0.62 mmol) 4-methoxyphenylboronic acid (0.14 g; 0.93 mmol), K_3_PO_4_ (0.26 g; 1.24 mmol) and Pd(PPh_3_)_4_ (72 mg; 0.062 mmol) as yellowish solid (115 mg; 61%). Mp 162–163 C. IR (film, cm^−1^) *ν*_max_= 1395 (S = O), 1375 (S = O), 1179 (S = O), 1156 (S = O). ^1^H NMR (400 MHz, DMSO-d_6_) δ = 3.80 (s, 3H), 4.50 (dd, 2H, *J* = 5.8, 1.0 Hz), 5.98–6.06 (m, 1H), 6.97 (d, 1H, *J* = 11.2 Hz), 7.02–7.07 (m, 2H), 7.38 (d, 1H, *J* = 8.4 Hz), 7.62–7.67 (m, 2H), 7.69 (dd, 1H, *J* = 8.4, 2.4 Hz), 7.73 (d, 1H, *J* = 2.4 Hz) ppm. ^13 ^C NMR (100 MHz, DMSO-d_6_) δ = 51.6, 55.2, 114.5, 120.5, 122.7, 127.9, 128.0, 128.4, 129.0, 130.8, 131.2, 138.7, 145.8, 159.3 ppm. Anal. Calcd for C_16_H_14_O_4_S: C, 63.56; H, 4.67. Found: C, 63.38; H, 4.68.

#### 7–(4-Fluorophenyl)-3*H*-1,2-benzoxathiepine 2,2-dioxide (10)


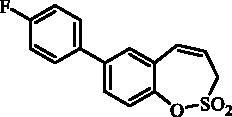
Compound **10** was prepared according to the general procedure from 7-iodo-3*H*-1,2-benzoxathiepine 2,2-dioxide (**7**) (0.20 g; 0.62 mmol) (4-fluorophenyl)boronic acid (0.13 g; 0.93 mmol), K_3_PO_4_ (0.26 g; 1.24 mmol) and Pd(PPh_3_)_4_ (72 mg; 0.062 mmol) as white solid (79 mg; 44%). Mp 117–118 °C. IR (film, cm^−1^) *ν*_max_=1373 (S = O), 1181 (S = O), 1168 (S = O). ^1^H NMR (400 MHz, CDCl_3_) δ = 4.06 (dd, 2H, *J* = 6.2, 1.2 Hz), 5.99–6.06 (m, 1H), 6.93 (d, 1H, *J* = 11.0 Hz), 7.12–7.18 (m, 2H), 7.39 (d, 1H, *J* = 8.4 Hz), 7.45 (d, 1H, *J* = 2.3 Hz), 7.49–7.55 (m, 2H), 7.57 (dd, 1H, *J* = 8.4, 2.3 Hz) ppm. ^13 ^C NMR (100 MHz, CDCl_3_) δ = 51.4, 116.1 (d, *J* = 21.6 Hz), 119.9, 123.4, 128.6, 128.9, 129.0, 129.2, 129.3, 132.8, 135.6 (d, *J* = 3.4 Hz), 139.6, 147.1, 163.0 (d, *J* = 247.0 Hz) ppm. Anal. Calcd for C_15_H_11_FO_3_S: C, 62.06; H, 3.82. Found: C, 62.34; H, 3.83.

#### 7–(4-(Trifluoromethyl)phenyl)-3*H*-1,2-benzoxathiepine 2,2-dioxide (11)


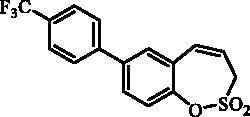
Compound **11** was prepared according to the general procedure from 7-iodo-3*H*-1,2-benzoxathiepine 2,2-dioxide (**7**) (0.20 g; 0.62 mmol) (4-(trifluoromethyl)phenyl)boronic acid (0.18 g; 0.93 mmol), K_3_PO_4_ (0.26 g; 1.24 mmol) and Pd(PPh_3_)_4_ (72 mg; 0.062 mmol) as white solid (140 mg; 66%). Mp 166–168 °C.

IR (film, cm^−1^) *ν*_max_=1357 (S = O), 1332 (S = O), 1166 (S = O). ^1^H NMR (400 MHz, DMSO-d_6_) δ = 4.56 (dd, 2H, *J* = 5.8, 1.0 Hz), 6.00–6.08 (m, 1H), 6.99 (d, 1H, *J* = 11.4 Hz), 7.47 (d, 1H, *J* = 8.4 Hz), 7.81–7.86 (m, 3H), 7.88 (d, 1H, *J* = 2.2 Hz), 7.94 (d, 2H, *J* = 8.2 Hz) ppm. ^13 ^C NMR (100 MHz, DMSO-d_6_) δ = 51.8, 120.8, 123.0, 124.3 (q, *J* = 273.0 Hz), 125.9 (q, *J* = 3.7 Hz), 127.7, 128.3 (q, *J* = 32.0 Hz), 128.6, 128.9, 130.3, 130.8, 137.4, 142.5, 146.9 ppm. Anal. Calcd for C_16_H_11_F_3_O_3_S: C, 56.47; H, 3.26. Found: C, 56.46; H, 3.28.

#### 7–(4-(Ethoxycarbonyl)phenyl)-3*H*-1,2-benzoxathiepine 2,2-dioxide (12)


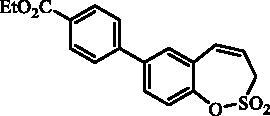
Compound **12** was prepared according to the general procedure from 7-iodo-3*H*-1,2-benzoxathiepine 2,2-dioxide (**7**) (0.20 g; 0.62 mmol) (4-(ethoxycarbonyl)phenyl)boronic acid (0.18 g; 0.93 mmol), K_3_PO_4_ (0.26 g; 1.24 mmol) and Pd(PPh_3_)_4_ (72 mg; 0.062 mmol) as yellowish solid (96 mg; 44%). Mp 141–142 °C. IR (film, cm^−1^) *ν*_max_= 1701 (C = O), 1380 (S = O), 1184 (S = O), 1170 (S = O). ^1^H NMR (400 MHz, DMSO-d_6_) δ = 1.34 (t, 3H, *J* = 7.1 Hz), 4.34 (q, 2H, *J* = 7.1 Hz), 4.55 (dd, 2H, *J* = 5.8, 1.2 Hz), 6.00–6.08 (m, 1H), 6.99 (d, 1H, *J* = 11.5 Hz), 7.46 (d, 1H, *J* = 8.5 Hz), 7.83 (dd, 1H, *J* = 8.5, 2.3 Hz), 7.85–7.90 (m, 3H), 8.03–8.08 (m, 2H) ppm. ^13 ^C NMR (100 MHz, DMSO-d_6_) δ = 14.2, 51.7, 60.8, 120.8, 123.0, 127.1, 128.6, 128.8, 129.2, 129.8, 130.1, 130.8, 137.7, 142.9, 146.9, 165.4 ppm. Anal. Calcd for C_18_H_16_O_5_S: C, 62.78; H, 4.68. Found: C, 62.76; H, 4.71.

#### 8-Phenyl-3*H*-1,2-benzoxathiepine 2,2-dioxide (21)


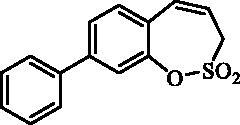
Compound **21** was prepared according to the general procedure from 8-bromo-3*H*-1,2-benzoxathiepine 2,2-dioxide (**20**) (0.25 g; 0.91 mmol) phenylboronic acid (0.17 g; 1.36 mmol), K_3_PO_4_ (0.39 g; 1.82 mmol) and Pd(PPh_3_)_4_ (105 mg; 0.091 mmol) as yellowish solid (109 mg; 44%). Mp 103–104 °C. IR (film, cm^−1^) *ν*_max_= 1376 (S = O), 1177 (S = O). ^1^H NMR (400 MHz, CDCl_3_) δ = 4.08 (dd, 2H, *J* = 6.1, 1.2 Hz), 5.94–6.01 (m, 1H), 6.88–6.93 (m, 1H), 7.36–7.43 (m, 2H), 7.44–7.50 (m, 2H), 7.55–7.63 (m, 4H) ppm. ^13 ^C KMR (100 MHz, CDCl_3_) δ = 51.6, 119.2, 121.3, 125.7, 126.8, 127.2, 128.5, 129.2, 131.3, 132.5, 138.9, 144.0, 148.1 ppm. Anal. Calcd for C_15_H_12_O_3_S: C, 66.16; H, 4.44. Found: C, 66.15; H, 4.46.

#### 8–(4-Methoxyphenyl)-3*H*-1,2-benzoxathiepine 2,2-dioxide (22)


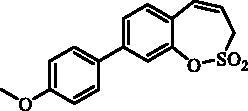
Compound **22** was prepared according to the general procedure from 8-bromo-3*H*-1,2-benzoxathiepine 2,2-dioxide (**20**) (0.25 g; 0.91 mmol) 4-methoxyphenylboronic acid (0.21 g; 1.36 mmol), K_3_PO_4_ (0.39 g; 1.82 mmol) and Pd(PPh_3_)_4_ (105 mg; 0.091 mmol) as yellowish solid (121 mg; 44%). Mp 142–143 °C. IR (film, cm^−1^) *ν*_max_= 1369 (S = O), 1177 (S = O), 1164 (S = O). ^1^H NMR (400 MHz, CDCl_3_) δ = 3.86 (s, 3H), 4.07 (dd, 2H, *J* = 6.1, 1.1 Hz), 5.92–5.99 (m, 1H), 6.88 (d, 1H, *J* = 11.1 Hz), 6.97–7.02 (m, 2H), 7.32–7.36 (m, 1H), 7.50–7.58 (m, 4H) ppm. ^13 ^C NMR (100 MHz, CDCl_3_) δ = 51.6, 55.5, 114.6, 118.8, 120.6, 125.2, 126.1, 128.3, 131.3, 132.6, 143.6, 148.2, 160.1 ppm. Anal. Calcd for C_16_H_14_O_4_S: C, 63.56; H, 4.67. Found: C, 63.20; H, 4.69.

#### 8–(4-Fluorophenyl)-3*H*-1,2-benzoxathiepine 2,2-dioxide (23)


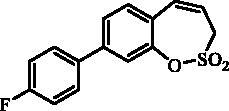
Compound **23** was prepared according to the general procedure from 8-bromo-3*H*-1,2-benzoxathiepine 2,2-dioxide (**20**) (0.25 g; 0.91 mmol) (4-fluorophenyl)boronic acid (0.19 g; 1.36 mmol), K_3_PO_4_ (0.39 g; 1.82 mmol) and Pd(PPh_3_)_4_ (105 mg; 0.091 mmol) as white solid (108 mg; 41%). Mp 111–112 °C. IR (film, cm^−1^) *ν*_max_= 1371 (S = O), 1168 (S = O). ^1^H NMR (400 MHz, CDCl_3_) δ = 4.08 (dd, 2H, *J* = 6.1, 1.2 Hz), 5.94–6.01 (m, 1H), 6.90 (d, 1H, *J* = 11.0 Hz), 7.12–7.19 (m, 2H), 7.35–7.40 (m, 1H), 7.50–7.53 (m, 2H), 7.54–7.60 (m, 2H) ppm. ^13 ^C NMR (100 MHz, CDCl_3_) δ = 51.7, 116.2 (d, *J* = 21.6 Hz), 119.3, 121.2, 125.6, 126.9, 128.9, 129.0, 131.5, 132.4, 135.0 (d, *J* = 3.3 Hz), 142.9, 148.1, 163.2 (d, *J* = 248.0 Hz) ppm. Anal. Calcd for C_15_H_11_FO_3_S: C, 62.06; H, 3.82. Found: C, 62.04; H, 3.86.

#### 8–(4-(Trifluoromethyl)phenyl)-3*H*-1,2-benzoxathiepine 2,2-dioxide (24)


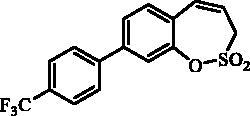
Compound **24** was prepared according to the general procedure from 8-bromo-3*H*-1,2-benzoxathiepine 2,2-dioxide (**20**) (0.25 g; 0.91 mmol) (4-(trifluoromethyl)phenyl)boronic acid (0.26 g; 1.36 mmol), K_3_PO_4_ (0.39 g; 1.82 mmol) and Pd(PPh_3_)_4_ (105 mg; 0.091 mmol) as white solid (142 mg; 46%). Mp 121–122 °C. IR (film, cm^−1^) *ν*_max_= 1366 (S = O), 1324 (S = O), 1172 (S = O). ^1^H NMR (400 MHz, CDCl_3_) δ = 4.11 (dd, 2H, *J* = 6.1, 1.2 Hz), 5.97–6.04 (m, 1H), 6.90 (d, 1H, *J* = 11.2 Hz), 7.40–7.44 (m, 1H), 7.55–7.60 (m, 2H), 7.70–7.75 (m, 4H) ppm. ^13 ^C NMR (100 MHz, CDCl_3_) δ = 51.8, 119.7, 121.6, 124.2 (q, *J* = 273.0 Hz), 125.9, 126.2 (q, *J* = 3.8 Hz), 127.6, 127.8, 130.5 (q, *J* = 32.9 Hz), 131.7, 132.2, 142.3, 142.4, 148.1 ppm. Anal. Calcd for C_16_H_11_F_3_O_3_S: C, 56.47; H, 3.26. Found: C, 56.23; H, 3.23.

#### 8–(4-(Ethoxycarbonyl)phenyl)-3*H*-1,2-benzoxathiepine 2,2-dioxide (25)


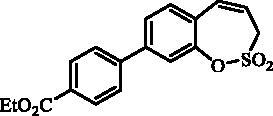
Compound **25** was prepared according to the general procedure from 8-bromo-3*H*-1,2-benzoxathiepine 2,2-dioxide (**20**) (0.25 g; 0.91 mmol) (4-(ethoxycarbonyl)phenyl)boronic acid (0.26 g; 1.36 mmol), K_3_PO_4_ (0.39 g; 1.82 mmol) and Pd(PPh_3_)_4_ (105 mg; 0.091 mmol) as white solid (119 mg; 38%). Mp 151–152 °C. IR (film, cm^−1^) *ν*_max_= 1703 (C = O), 1366 (S = O), 1175 (S = O). ^1^H NMR (400 MHz, CDCl_3_) δ = 1.42 (t, 3H, *J* = 7.1 Hz), 4.10 (dd, 2H, *J* = 6.1, 1.2 Hz), 4.41 (q, 2H, *J* = 7.1 Hz), 5.96–6.03 (m, 1H), 6.90 (d, 1H, *J* = 11.2 Hz), 7.39–7.43 (m, 1H), 7.57–7.62 (m, 2H), 7.65–7.70 (m, 2H), 8.11–8.16 (m, 2H) ppm. ^13 ^C NMR (100 MHz, CDCl_3_) δ = 14.5, 51.7, 61.3, 119.6, 121.5, 125.9, 127.1, 127.7, 130.4, 131.6, 132.3, 142.7, 143.0, 148.1, 166.3 ppm. Anal. Calcd for C_18_H_16_O_5_S: C, 62.78; H, 4.68. Found: C, 62.50; H, 4.70.

#### 9- Phenyl-3*H*-1,2-benzoxathiepine 2,2-dioxide (30)


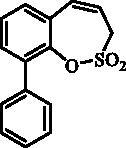
Compound **30** was prepared according to the general procedure from 9-bromo-3*H*-1,2-benzoxathiepine 2,2-dioxide (**29**) (0.25 g; 0.91 mmol) phenylboronic acid (0.17 g; 1.36 mmol), K_3_PO_4_ (0.39 g; 1.82 mmol) and Pd(PPh_3_)_4_ (105 mg; 0.091 mmol) as white solid (104 mg; 42%). Mp 135–136 °C. IR (film, cm^−1^) *ν*_max_= 1370 (S = O), 1162 (S = O). ^1^H NMR (400 MHz, CDCl_3_) δ = 4.08 (dd, 2H, *J* = 5.8, 1.3 Hz), 5.87–5.94 (m, 1H), 6.85–6.90 (m, 1H), 7.29 (dd, 1H, *J* = 7.6, 1.8 Hz), 7.35–7.42 (m, 2H), 7.43–7.49 (m, 3H), 7.51–7.55 (m, 2H) ppm. ^13 ^C KMR (100 MHz, CDCl_3_) δ = 52.1, 118.9, 127.1, 128.1, 128.5, 128.6, 129.6, 130.5, 132.1, 132.5, 136.3, 136.5, 144.7 ppm. Anal. Calcd for C_15_H_12_O_3_S: C, 66.16; H, 4.44. Found: C, 66.15; H, 4.46.

#### 9–(4-Methoxyphenyl)-3*H*-1,2-benzoxathiepine 2,2-dioxide (31)


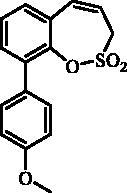
Compound **31** was prepared according to the general procedure from 9-bromo-3*H*-1,2-benzoxathiepine 2,2-dioxide (**29**) (0.25 g; 0.91 mmol) 4-methoxyphenylboronic acid (0.21 g; 1.36 mmol), K_3_PO_4_ (0.39 g; 1.82 mmol) and Pd(PPh_3_)_4_ (105 mg; 0.091 mmol) as white solid (110 mg; 40%). Mp 113–114 °C. IR (film, cm^−1^) *ν*_max_= 1369 (S = O), 1181 (S = O), 1154 (S = O). ^1^H NMR (400 MHz, CDCl_3_) δ = 3.85 (s, 3H), 4.08 (dd, 2H, *J* = 5.8, 1.3 Hz), 5.86–5.94 (m, 1H), 6.84–6.89 (m, 1H), 6.97–7.02 (m, 2H), 7.23–7.27 (m, 1H), 7.34 (t, 1H, *J* = 7.6 Hz), 7.42 (dd, 1H, *J* = 7.6, 1.8 Hz), 7.45–7.50 (m, 2H) ppm. ^13 ^C NMR (100 MHz, CDCl_3_) δ = 52.0, 55.4, 114.0, 118.9, 127.1, 128.6, 128.7, 130.1, 130.8, 132.0, 132.6, 136.2, 144.7, 159.5 ppm. Anal. Calcd for C_16_H_14_O_4_S: C, 63.56; H, 4.67. Found: C, 63.58; H, 4.70.

#### 9–(4-Fluorophenyl)-3*H*-1,2-benzoxathiepine 2,2-dioxide (32)


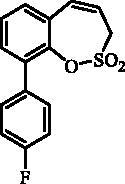
Compound **32** was prepared according to the general procedure from 9-bromo-3*H*-1,2-benzoxathiepine 2,2-dioxide (**29**) (0.25 g; 0.91 mmol) (4-fluorophenyl)boronic acid (0.19 g; 1.36 mmol), K_3_PO_4_ (0.39 g; 1.82 mmol) and Pd(PPh_3_)_4_ (105 mg; 0.091 mmol) as white solid (103 mg; 39%). Mp 130–131 °C. IR (film, cm^−1^) *ν*_max_=1370 (S = O), 1154 (S = O). ^1^H NMR (400 MHz, CDCl_3_) δ = 4.08 (dd, 2H, *J* = 5.8, 1.3 Hz), 5.88–5.95 (m, 1H), 6.85–6.90 (m, 1H), 7.10–7.18 (m, 2H), 7.30 (dd, 1H, *J* = 7.5, 2.0 Hz), 7.37 (t, 1H, *J* = 7.5 Hz), 7.41 (dd, 1H, *J* = 7.5, 2.0 Hz), 7.47–7.53 (m, 2H) ppm. ^13 ^C NMR (100 MHz, CDCl_3_) δ = 52.1, 115.5 (d, *J* = 21.6 Hz), 119.1, 127.2, 128.7, 130.6, 131.3, 131.4, 132.0, 132.3 (d, *J* = 3.3 Hz), 132.5, 135.6, 144.7, 162.8 (d, *J* = 247.0 Hz) ppm. Anal. Calcd for C_15_H_11_FO_3_S: C, 62.06; H, 3.82. Found: C, 62.05; H, 3.84.

#### 9–(4-(Trifluoromethyl)phenyl)-3*H*-1,2-benzoxathiepine 2,2-dioxide (33)


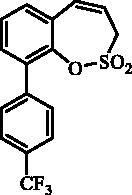
Compound **33** was prepared according to the general procedure from 9-bromo-3*H*-1,2-benzoxathiepine 2,2-dioxide (**29**) (0.25 g; 0.91 mmol) (4-(trifluoromethyl)phenyl)boronic acid (0.26 g; 1.36 mmol), K_3_PO_4_ (0.39 g; 1.82 mmol) and Pd(PPh_3_)_4_ (105 mg; 0.091 mmol) as white solid (136 mg; 44%). Mp 115–116 °C.

IR (film, cm^−1^) *ν*_max_= 1333 (S = O), 1166 (S = O). ^1^H NMR (400 MHz, CDCl_3_) δ = 4.10 (dd, 2H, *J* = 5.8, 1.3 Hz), 5.90–5.97 (m, 1H), 6.86–6.91 (m, 1H), 7.35 (dd, 1H, *J* = 7.0, 2.6 Hz), 7.38–7.45 (m, 2H), 7.62–7.67 (m, 2H), 7.70–7.74 (m, 2H) ppm. ^13 ^C NMR (100 MHz, CDCl_3_) δ = 52.2, 119.2, 124.5 (q, *J* = 273.0 Hz), 125.5 (q, *J* = 3.8 Hz), 127.3, 128.9, 130.0, 130.2 (q, *J* = 32.0 Hz), 131.3, 131.9, 132.3, 135.2, 140.0 (q, *J* = 1.5 Hz), 144.6 ppm. Anal. Calcd for C_16_H_11_F_3_O_3_S: C, 56.47; H, 3.26. Found: C, 56.21; H, 3.29.

#### 9–(4-(Ethoxycarbonyl)phenyl)-3*H*-1,2-benzoxathiepine 2,2-dioxide (34)


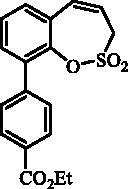
Compound **34** was prepared according to the general procedure from 9-bromo-3*H*-1,2-benzoxathiepine 2,2-dioxide (**29**) (0.25 g; 0.91 mmol) (4-(ethoxycarbonyl)phenyl)boronic acid (0.26 g; 1.36 mmol), K_3_PO_4_ (0.39 g; 1.82 mmol) and Pd(PPh_3_)_4_ (105 mg; 0.091 mmol) as white solid (113 mg; 36%). Mp 105–106 °C.

IR (film, cm^−1^) *ν*_max_= 1714 (C = O), 1375 (S = O), 1157 (S = O). ^1^H NMR (400 MHz, CDCl_3_) δ = 1.41 (t, 3H, *J* = 7.1 Hz), 4.08 (dd, 2H, *J* = 5.8, 1.1 Hz), 4.40 (q, 2H, *J* = 7.1 Hz), 5.89–5.97 (m, 1H), 6.88 (d, 1H, *J* = 11.4 Hz), 7.31–7.46 (m, 3H), 7.58–7.63 (m, 2H), 8.11–8.16 (m, 2H) ppm. ^13 ^C NMR (100 MHz, CDCl_3_) δ = 14.5, 52.1, 61.1, 119.2, 127.2, 128.8, 129.6, 129.7, 130.1, 131.1, 131.8, 132.4, 135.6, 140.9, 144.6, 166.5 ppm. Anal. Calcd for C_18_H_16_O_5_S: C, 62.78; H, 4.68. Found: C, 62.28; H, 4.69.

### 2.2. CA inhibitory assay

An Applied Photophysics stopped-flow instrument has been used for assaying the CA catalysed CO_2_ hydration activity^15^. Phenol red (at a concentration of 0.2 mM) was used as indicator, working at the absorbance maximum of 557 nm, with 20 mM Hepes (pH 7.5) as buffer and 20 mM Na_2_SO_4_ (for maintaining constant the ionic strength), following the initial rates of the CA-catalysed CO_2_ hydration reaction for a period of 10 − 100 s. The CO_2_ concentrations ranged from 1.7 to 17 mM for the determination of the kinetic parameters and inhibition constants. For each inhibitor, at least six traces of the initial 5 − 10% of the reaction have been used for determining the initial velocity. The uncatalysed rates were determined in the same manner and subtracted from the total observed rates. Stock solutions of inhibitor (0.1 mM) were prepared in distilled − deionised water, and dilutions up to 0.01 nM were done thereafter with the assay buffer. Inhibitor and enzyme solutions were preincubated together for 6 h at room temperature prior to assay in order to allow for the formation of the E − I complex. The inhibition constants were obtained by nonlinear least-squares methods using PRISM 3 and the Cheng − Prusoff equation, as reported earlier^16–19^, and represent the mean from at least three different determinations. All CA isoforms were recombinant ones obtained in-house as reported earlier^19^^,^^20^.

## 3. Results and discussion

### 3.1. Chemistry

**Scheme 1.**
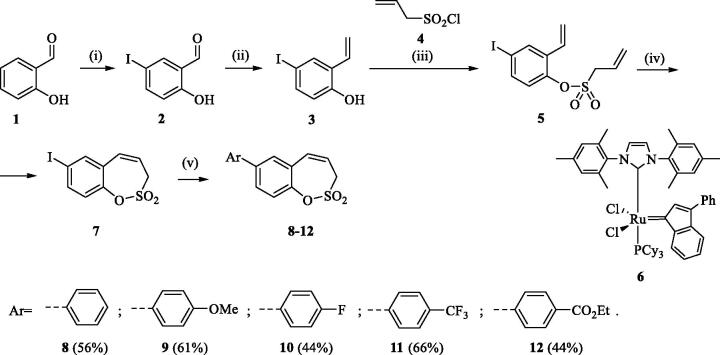
Reagents and conditions for the preparation of derivatives **8–12**: (i) ICl, AcOH, 40 °C, 24 h, 84%; (ii) KOtBu, CH_3_P(C_6_H_5_)_3_Br, THF, RT, 18 h, 83%; (iii) NEt_3_, CH_2_Cl_2_, 0 °C to RT, 4 h, 83%; (iv) toluene, 70 °C, 4 h, 89%; (v) Ar-B(OH)_2_, Pd(PPh_3_)_4_, K_3_PO_4_, toluene/H_2_O, 100 °C, 16 h.

The synthesis of desired compounds is partly based on the strategy previously developed by our groups^10^. The synthesis of 7-aryl 3H-1,2-benzoxathiepine 2,2-dioxides starts with the iodination of salicylaldehyde (**1**) by iodine monochloride and corresponding iodo derivative **2** was isolated in good yield (Scheme 1)^11^. Under Wittig reaction conditions aldehyde **2** was converted to olefin **3**, which was treated by sulphonyl chloride **4** thus providing bis-olefin **5** in 83% yield. To obtain the key intermediate **7,** the ring closure in compound **5** was performed in olefin metathesis conditions, using Ru-catalyst **6**. The key intermediate **7** was reacted with a series of aryl boronic acids under Suzuki reaction conditions and the desired 7-aryl 3H-1,2-benzoxathiepine 2,2-dioxides **8**–**12** were isolated in acceptable yields (44–66%) (Scheme 1).

**Scheme 2.**
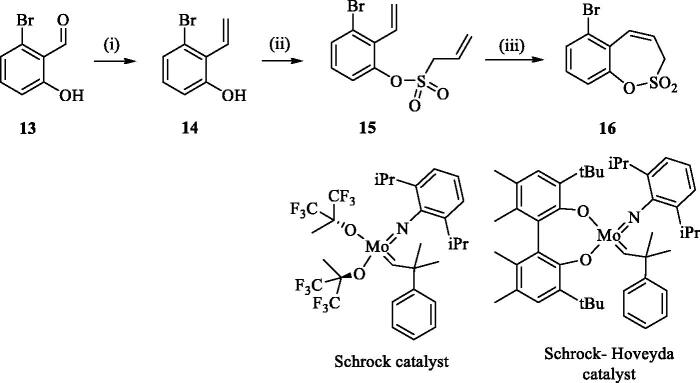
Reagents and conditions: (i) KOtBu, CH_3_P(C_6_H_5_)_3_Br, THF, RT, 18 h, 82%; (ii) **4**, NEt_3_, CH_2_Cl_2_, 0 °C to RT, 4 h, 66%; (iii) a) **6** (5 mol% and 10 mol%), toluene, 70 °C, 40 h, 0%; b) Schrock catalyst [Mo] (10 mol%), toluene, 70 °C, 16 h, 0%; c) Schrock–Hoveyda [Mo] (10 mol%), toluene, 70 °C, 16 h, 0%;

In an attempt to prepare 6-aryl 3H-1,2-benzoxathiepine 2,2-dioxides, the commercially available bromo salicylaldehyde **13** was first converted to olefin **14** under Wittig reaction conditions, followed by treatment with sulphonyl chloride **4**, thus providing bis-olefin **15** for olefin metathesis ring closure reaction (Scheme 2). Utilisation of the Ru-catalyst **6** as described above did not provide the formation of the desired key intermediate 6-bromo 3H-1,2-benzoxathiepine 2,2-dioxide (**16**) even at prolonged reaction times. By doubling catalyst **6** amount (10 mol%) only traces of compound **16** were observed after 40 h. No product formation was observed also when using Schrock and Schrock–Hoveyda Mo-catalysts. Probably olefin metathesis ring closure reaction did not take place due to sterical constraints due to the bulky Br atom at 3-positon of bis-olefin **15**.

**Scheme 3.**
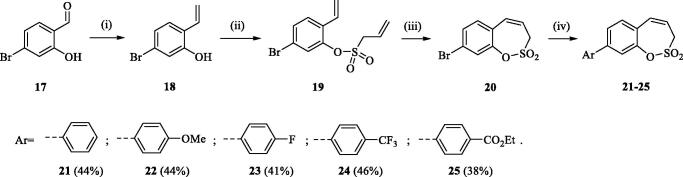
Reagents and conditions: (i) KOtBu, CH_3_P(C_6_H_5_)_3_Br, THF, RT, 18 h, 76%; (ii) **4**, NEt_3_, CH_2_Cl_2_, 0 °C to RT, 4 h, 54%; (iii) **6**, toluene, 70 °C, 4 h, 90%; (iv) Ar-B(OH)_2_, Pd(PPh_3_)_4_, K_3_PO_4_, toluene/H_2_O, 100 °C, 16 h.

The synthesis of 8-bromo intermediate **20** was started from commercially available aldehyde **17**, when under Wittig reaction conditions olefin **18** was obtained, which was thereafter treated with sulphonyl chloride **4** and provided the bis-olefin **19** in good yield (Scheme 3). Ru-catalysed olefin metathesis afforded the key intermediate **20** which in turn, by reaction with a series of aryl boronic acids under Suzuki reaction condition, provided the desired compounds **21**–**25**.

**Scheme 4.**
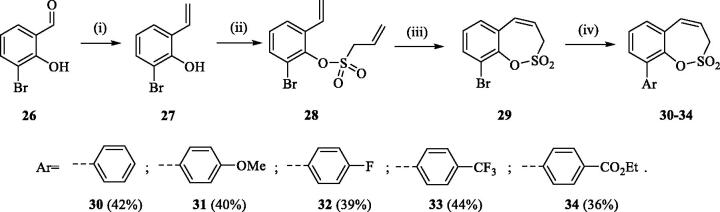
Reagents and conditions: (i) KOtBu, CH_3_P(C_6_H_5_)_3_Br, THF, RT, 18 h, 80%; (ii) **4**, NEt_3_, CH_2_Cl_2_, 0 °C to RT, 4 h, 86%; (iii) **6**, toluene, 70 °C, 4 h, 78%; (iv) Ar-B(OH)_2_, Pd(PPh_3_)_4_, K_3_PO_4_, toluene/H_2_O, 100 °C, 16 h.

The same strategy was successfully utilised for the synthesis of a series of 9-aryl 3H-1,2-benzoxathiepine 2,2-dioxides starting by the treatment of aldehyde **26** with methyltriphenylphosphonium bromide under Wittig reaction conditions (Scheme 4). The obtained phenol **27** was reacted with sulphonyl chloride **4** and ring closure of isolated **28** was successfully performed in Ru-catalysed olefin metathesis conditions, providing bromide **29**. Further reaction of compound **9** with aryl boronic acids provided the desired derivatives **30**–**34** in moderate yields.

### 3.2. Carbonic anhydrase inhibition

The obtained homosulfocoumarins **7–34** were investigated for their CA inhibitory properties by using a stopped-flow CO_2_ hydrase assay^15^ and four human CA isoforms (hCA I, II, IX and XII) known to be drug targets^1^ (Table 1).

**Table 1.** Inhibition data of human CA isoforms CA I, II, IX and XII with 3H-1,2-benzoxathiepines 2,2-dioxide **7–34** using **AAZ** as a standard drug.
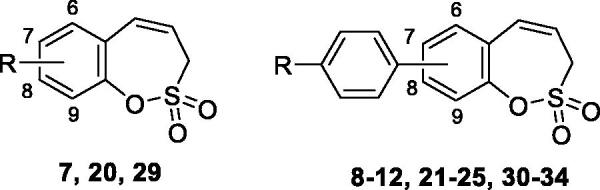

Cmpd	7 / 8 / 9	R	*K*_I_ (nM)*			
			CA I	CA II	CA IX	CA XII
**7**	7	I	>100 µM	>100 µM	66.2	455.5
**8**	7	H	>100 µM	>100 µM	654.8	1376
**9**	7	OCH_3_	>100 µM	>100 µM	407.6	2934
**10**	7	F	>100 µM	>100 µM	330.8	890.5
**11**	7	CF_3_	>100 µM	>100 µM	221.4	4017
**12**	7	CO_2_CH_2_CH_3_	>100 µM	>100 µM	620.8	2398
**20**	8	Br	>100 µM	>100 µM	47.5	132.9
**21**	8	H	>100 µM	>100 µM	104.8	473.2
**22**	8	OCH_3_	>100 µM	>100 µM	63.1	168.6
**23**	8	F	>100 µM	>100 µM	95.2	77.9
**24**	8	CF_3_	>100 µM	>100 µM	44.0	247.8
**25**	8	CO_2_CH_2_CH_3_	>100 µM	>100 µM	79.8	289.3
**29**	9	Br	>100 µM	>100 µM	754.8	3824
**30**	9	H	>100 µM	>100 µM	21.1 µM	>100 µM
**31**	9	OCH_3_	>100 µM	>100 µM	60.9 µM	>100 µM
**32**	9	F	>100 µM	>100 µM	33.7 µM	>100 µM
**33**	9	CF_3_	>100 µM	>100 µM	47.1 µM	>100 µM
**34**	9	CO_2_CH_2_CH_3_	>100 µM	>100 µM	16.4 µM	>100 µM
**AAZ**	–	–	250	12	25	5.7

^*^ Mean from three different assays, by a stopped flow technique (errors were in the range of ± 5–10% of the reported values).

The following structure-activity relationship (SAR) can be observed from the inhibition data of Table 1.

(i) as the previously reported homosulfocoumarins^10^ and similar to sulfocoumarins^7–9^, also the derivatives reported here did not significantly inhibit the cytosolic isoforms hCA I and II, unlike the sulphonamide acetazolamide (used as standard CAI), which has a very good affinity (in the nanomolar range) for hCA II and a micromolar one for hCA I (Table 1).

(ii) the transmembrane, tumour-associated isoforms hCA IX and XII were effectively inhibited by derivatives **7–29** reported here (in the low – medium nanomolar arneg) and were poorly inhibited, in the micromolar range by the 9-substituted-homosulfocoumarins 30–34 (K_I_s in the range of 16.4–60.9 µM against hCA IX and >100 µM against hCA XII). Thus, although weak inhibitors, these sulfocoumarins are anyhow highly selective for the inhibition of hCA IX, whereas their activity against hCA I, II and XII is absent (Table 1). As already anticipated above, the most important factors associated with CA IX/XII inhibitory activity are the position and the nature of the moieties present on the six-membered ring of the homosulfocoumarin. Indeed, for 9-substituted derivatives, the presence of bulky, substituted aryls as in **30–34** leads to low activity, as mentioned above. Only the 9-bromo-derivative **29** had a medium potency inhibitory action against the two isoforms, with K_I_s in the range of 754.8 – 3824 nM. On the contrary, the 8-substituted derivatives **20–25** showed a much better inhibitory power against both isoforms, being generally more potent than the corresponding 7-substituted derivatives **7–12**. Indeed, for the 7-substituted homosulfocoumarins the K_I_s were in the range of 66.2 – 620.8 nM against hCA IX and of 455.5 – 2934 nM against hCA XII. On the contrary, for the 8- substituted homosulfocoumarins, the K_I_s were in the range of 44.0 – 104.8 nM against hCA IX and in the range of 77.9 – 473.2 nM for hCA XII (Table 1). The 8–(4-trifluoromethyl)phenyl-substituted homosulfocoumarin **24** was the most effective hCA IX inhibitor (potency in the same range as AAZ), whereas the corresponding 4-fluorophenyl derivative **23** was the best hCA XII inhibitor in the new series of compounds investigated here but it was an order of magnitude less effective compared to acetazolamide.

## 4. Conclusions

A new series of homosulfocoumarins (3H-1,2-benzoxathiepine 2,2-dioxides) possessing various moieties in the 7, 8 or 9 position of the heterocylic ring were prepared by original procedures and investigated for the inhibition of four physiologically relevant CA isoforms, hCA I, II, IX and XII. The 8-substituted homosulfocoumarins were the most effective hCA IX/XII inhibitors followed by the 7-substituted derivatives, whereas the substitution pattern in position 9 led to less effective inhibitors for these transmembrane, tumour-associated isoforms. The cytosolic isoforms hCA I and II were not inhibited by these compounds, similar to the sulfocoumarins/coumarins investigated earlier. As hCA IX and XII are validated anti-tumour targets^5^, with one sulphonamide (SLC-0111) in Phase Ib/II clinical trials, finding derivatives with a better selectivity for inhibiting the tumour-associated isoforms over the cytosolic ones, as the homosulfocoumarins reported here, is of crucial importance.
